# Depression Affects Intrinsic Brain Activity in Patients With Mild Cognitive Impairment

**DOI:** 10.3389/fnins.2019.01333

**Published:** 2019-12-17

**Authors:** Yang Yu, Ziqi Li, Yajie Lin, Jie Yu, Guoping Peng, Kan Zhang, Xize Jia, Benyan Luo

**Affiliations:** ^1^Department of Neurology, First Affiliated Hospital, School of Medicine, Zhejiang University, Hangzhou, China; ^2^School of Information and Electronics Technology, Jiamusi University, Jiamusi, China; ^3^Center for Cognition and Brain Disorders, Institutes of Psychological Sciences, Hangzhou Normal University, Hangzhou, China; ^4^Zhejiang Key Laboratory for Research in Assessment of Cognitive Impairments, Hangzhou, China

**Keywords:** Alzheimer’s disease, aging, cognition, depression, brain function

## Abstract

Numerous observational studies have shown that depressive symptoms are common in individuals with mild cognitive impairment (MCI) who have a higher rate of progress to dementia. However, it is still uncertain whether there are any differences between MCI patients with and without depression symptom in their brain function activities. Here we have identified the brain function activity differences in two groups of MCI patients (with depression or without depression) using the resting state MRI (rsfMRI) measurements. 76 right-handed MCI subjects have been recruited in this study, including 27 MCI patients with depression symptom (MCID), 49 MCI patients without depression symptom (MCIND). Analyses based on 7 rsfMRI measurements, including four static measurements (ALFF, fALFF, PerAF, and ReHo) and three dynamic measurements (dALFF, dfALFF, and dReHo) have been used to explore the temporal variability of intrinsic brain activity. No significant differences in ALFF and dALFF between the two group were found. In the MCID group, fALFF decreased in temporal gyrus, frontal gyrus, inferior occipital gyrus, middle frontal gyrus and cerebellum, but increased in cuneus, calcarine, lingual; while PerAF increased in left parahippocampus. The differences of ReHo in the two groups was only found in cerebellum. Compared to MCIND group, dfALFF in MCID decreased in cuneus, occipital gyrus and calcarine, while dReHo in MCID increased in bilateral temporal gyrus, frontal gyrus, superior parietal gyrus, inferior parietal gyrus and precuneus. Our results may provide a better understanding in the relationship between the depressive symptoms and memory deficits.

## Introduction

Mild cognitive impairment (MCI) is the clinical status of an individual with memory impairment who had memory defect but is otherwise functioning properly and does not meet clinical diagnosis criteria for dementia (Petersen et al., 1999, 2001). Cognitive deficits can be a single symptom, they also coexist with other non-cognitive features, of all the non-cognitive features, the prevalence of depression is the highest (Chi et al., 2015). Further, evidence suggests that MCI patients combined with depression symptoms (MCID) progress more rapidly from MCI to Alzheimer’s disease (AD) along the neurodegenerative spectrum, with a reported prevalence of 32% (Gao et al., 2013; Chi et al., 2015; Ismail et al., 2017). Thus, for early diagnosis and treatment purposes, an appropriate screening strategy to define the probable risk factors in those cognitive impaired individuals is meaningful (Ismail et al., 2017).

In recent years, based on the development of neuroimaging technology, magnetic resonance imaging, as a tool for detecting brain structure and function has been provided. Blood oxygenation level-dependent (BOLD) resting-state functional MRI (rsfMRI), has attracted enormous research interest in studying the neural mechanisms of cognitive dysfunction in individuals with psychiatric disorders (Biswal, 2012). Various rsfMRI measures such as functional connectivity (FC) (Biswal et al., 1995), amplitude of low-frequency fluctuation (ALFF) (Zang et al., 2007) fractional ALFF (fALFF) (Zou et al., 2008), percent amplitude of fluctuation (PerAF) (Jia et al., 2017, 2019) regional homogeneity (ReHo) (Zang et al., 2004) and degree centrality (DC) (Zuo et al., 2012) have been used to describe the intrinsic brain activity (IBA). IBA involves dynamic neural and metabolic activities, it is activity and plays a pivotal role in brain function (Dai and He, 2014; Raichle, 2015). These methods have been widely used to evaluate the IBA of neurological disorders or neuropsychiatric disorders, such as AD, depression, and MCI (Yao et al., 2009; Zhang et al., 2012; Guo et al., 2016; Wee et al., 2016; Li et al., 2017; Stoyanov et al., 2017; Zhang et al., 2017; Kandilarova et al., 2018; Yang et al., 2018; Liu et al., 2019). Furthermore, evidence indicated that once brain got an internal or external stimuli, it could respond by dynamic integration or adjustment over multiple time scales (Hutchison et al., 2013; Bassett and Sporns, 2017; Yan et al., 2017). However, the aforementioned measures are static, which ignoring the characteristics of dynamic changes of IBA over time, they assumed that during the entire rsfMRI scan, the BOLD signal is stationary (Liao et al., 2014, 2015). Compared with the static rsfMRI measures, the dynamic sliding window approaches are effective for capturing the dynamic characteristics of regional brain activity over different times which could be used to examine abnormal brain function (Yan et al., 2017; Tang et al., 2018). These evidence all indicated that rsfMRI is a proper approach to compare the differences between MCID and MCIND.

Studies have identified regions in MCI compared with the NC, with a decreased or increased ALFF/fALFF/ReHo (Han et al., 2011; Zhang et al., 2012; Dai and He, 2014; Li et al., 2017; Liu et al., 2018, 2019; Yang et al., 2018). Previous study found that changes from ALFF/fALFF measurements of IBA may be worthwhile to characterize the early and gradual changes in physiological alterations throughout AD progression (Yang et al., 2018). Moreover, in MCID group the FC density values were higher in the left MTG than those in the MCI without depression patients (MCIND) (Liu et al., 2018). Another study found that abnormal ALFF values in MCID group could serve as markers to effectively differentiate MCID from MCI patients (Li et al., 2017). As for ReHo, it can be used to classify the depression subtypes and MCI, also changes in ReHo could be a biomarker for the pathophysiology and therapeutic response of depression (Guo et al., 2013; Liu et al., 2019). Previously, by combining dynamic FC with static FC, some studies found that the diagnostic accuracy for MCI could be significantly improved (Wee et al., 2016; Zhang et al., 2017). So far, no study explored the dynamic characteristics of local brain activity indexes in MCID patients.

We employed 7 resting state measurements, including four static measurements (ALFF, fALFF, PerAF, and ReHo) and three dynamic measurements (dALFF, dfALFF, and dReHo) to investigate the temporal variability of voxel-wise brain activity. These combinations were designed to explore the variability of IBA and to enhance our understanding of brain function by recognizing specific pathophysiological features and further deepen our understanding of cognitive behavior. We assumed that MCID patients would exhibit abnormal spontaneous brain activity compared with those MCIND. These would enhance understanding of the relationship between depressive symptoms and memory deficits.

## Materials and Methods

### Participants

The study was endorsed by the Research Ethics Review Board of the First Affiliated Hospital of Medical School of Zhejiang University (FAHZU). A total of 76 right-handed MCI subjects were recruited in the study, including 27 patients combined with depression symptoms (MCID), 49 MCI without depression patients (MCIND). All MCI patients were recruited at the clinic of the Department of Neurology, FAHZU. Diagnoses of MCI were made by experienced neurologists according to Petersen’s criteria (Petersen, 2004). Depressive symptoms were identified by qualified psychiatrists according to the Diagnostic and Statistical Manual of Mental Disorders, fifth edition (DSM-V) and the Geriatric Depression Scale (GDS) (Yesavage et al., 1982; Chau et al., 2006) [we also recruited 50 right-handed normal controls, who were matched for age and gender from the local communities (Supplementary Methods in Supplementary Material)].

The diagnosis of MCID and MCIND both fulfilled the published MCI diagnostic criteria (Petersen, 2004). The inclusion criteria for the MCID group included an acute episode of mild depression with DSM-V for the diagnosis of depression symptom who were first suffered from MCI and the 30-item GDS was > 10 scores (Yesavage et al., 1982; Chau et al., 2006). The MCIND subjects were excluded if they had been diagnosed with major depression, recurrent depression, or other psychiatric disorders as described in DSM-V.

### Imaging Data Acquisition

MRI data were obtained using a 3.0 Tesla GE Discovery MR750 scanner (HD, General Electric Healthcare, Waukesha, WI, United States). 3D T1-weighted structural images were acquired using the following parameters: 128 slices, TR of 8,100 ms, TE of 3.1 ms, slice thickness of 1 mm, FA of 8°, matrix size of 256 × 256, FOV of 256 × 256 mm^2^. Functional images were acquired using the following parameters: 43 contiguous axial slices, repetition time (TR) of 2,000 ms, echo time (TE) of 30 ms, slice thickness of 3.2 mm, flip angle (FA) of 90°, matrix size of 64 × 64, field of view (FOV) of 200 × 200 mm^2^, total scan time of 8’00”. During the rsfMRI scan, the patients were given no task but were instructed to simply rest with eyes closed.

### rsfMRI Preprocessing

The rsfMRI data were processed using SPM12^1^ and RESTplus (Jia et al., 2019)^2^. The first 10 time points were discarded as adaptation of the participant to the scanner noise. The data preprocessing steps included slice timing, realignment, and spatial normalization. First, an individual T1-weighted image was co-registered to the mean functional image and then the T1-weighted image was segmented into gray matter (GM), white matter (WM) signal, and cerebrospinal fluid (CSF) signal. The EPI images were spatially normalized to the Montreal Neurological Institute (MNI) space and voxel size was resampled to 3 mm × 3 mm × 3 mm using the normalization parameters estimated during segmentation. Smoothing was performed with a 6 mm full width - half maximum (FWHM) Gaussian kernel. After removing the linear trend, we regressed out of covariates, which consisted of Friston-24 head motion parameters (Friston et al., 1996; Yan et al., 2013), WM signal, and CSF signal. The time courses were filtered by a (0.01–0.08 Hz) band to reduce high-frequency noise and low-frequency drifts.

### Static ALFF, Fractional ALFF (fALFF), Percent Amplitude of Fluctuation (PerAF) and Regional Homogeneity (ReHo) Calculation

We performed ALFF, fALFF, PerAF and ReHo analysis for each scan. The calculation of ALFF was based on fast Fourier transform (FFT). Using FFT, each time course was converted to the frequency domain. Then, the square root of the power spectrum at each frequency was averaged across the filtered band (0.01–0.08 Hz). The ALFF of each voxel was then normalized by the global mean of the ALFF values (mALFF) for standardization. For each given voxel, mALFF reflected the degree of its raw ALFF value relative to the average ALFF value of the whole brain (Zang et al., 2007). Then we calculated fALFF by obtaining the ratio of the power spectrum of low frequency (0.01–0.08 Hz) to that of the entire frequency range. Then, the resulting spatial fALFF maps were then divided with each voxel divided by the whole-brain fALFF mean (mfALFF), providing mfALFF spatial maps (Zou et al., 2008). PerAF designated the percentage amplitude of BOLD fluctuation relative to the mean BOLD signal intensity of a given time series (Jia et al., 2017) with RESTplus (Jia et al., 2019). PerAF is standardized at the single voxel level, the resulting spatial PerAF maps were then normalized with each voxel divided by the global mean PerAF (mPerAF). Both PerAF and mPerAF can be used for group-level statistical analysis, here we used mPerAF for further statistical analyses (Jia et al., 2019; Yu et al., 2019). For ReHo, the Kendall’s coefficient of concordance (KCC) of the time course of every 27 nearest neighboring voxels was calculated (Zang et al., 2004). To reduce the influence of individual variations in the KCC value, ReHo map normalizations were performed by dividing the KCC among each voxel by the averaged KCC of the whole brain.

### Dynamic ALFF, fALFF and ReHo Calculation

Dynamic parameters were performed using Temporal Dynamic Analysis (TDA) toolkits based on DPABI (Yan et al., 2016) Sliding window-based analysis, which is sensitive in detecting time-dependent variations, was applied to examine three dynamic measurements (dALFF, dfALFF or dReHo) variability over the whole brain (Hindriks et al., 2016; Liu et al., 2017; Yan et al., 2017; Yip et al., 2017; Tang et al., 2018; Vergara et al., 2019).

In the sliding window analysis, a temporal window of certain size and shape is chosen, and ALFF, fALFF and ReHo within that window are calculated. Theoretically, the window size should be designed feasibly. It should be small enough to monitor potentially transient signals, and yet large enough to describe the lowest frequencies of interest in the signals (Sakoglu et al., 2010). Previous studies of sliding window connectivity have applied a sliding window length from to 10 to 180 s (Thompson et al., 2013; Gonzalez-Castillo et al., 2015; Chen et al., 2018). Here we applied a sliding window length of 32 TR (64 s) and a shifting step size of two TR (4 s) (Chen et al., 2018). The remaining 230 time points after removing the first 10 time points for each individual were segmented into 100 windows in total. In each sliding window, ALFF, fALFF and ReHo were calculated. After calculating ALFF of all voxel in time windows, each participant will get several window-based ALFF maps (similar as fALFF and ReHo). Then, we computed the mean and standard deviation of each voxel in all window-based ALFF maps for each subject and further got the corresponding coefficient of variation (CV) which was acquired by dividing the standard deviation by the mean. To better measure the dynamic variation of regional brain activity between different individuals, we used CV as dALFF (similar as dfALFF), which represented the temporal variability of absolute energy consumption in low-frequency regional brain activity (Tang et al., 2018).

### Statistical Analyses

#### Scales Analysis

To examine the between-group differences in the seven measurements, two-sample *t*-test was held between the MCID and MCIND groups using DPABI (Yan et al., 2016). The figure was drawn by both DPABI and BrainNet Viewer (Xia et al., 2013; Yan et al., 2016). To reduce the effect of confounding variables in the statistical analysis, we performed two-sample *t*-tests with the mean relative displacements of age, sex, and education as covariance. Multiple comparison correction was performed based on Gaussian random field theory (GRF, voxel-wise *p* < 0.05, cluster-wise *p* < 0.05, two-tailed).

#### Correlation Analysis

With the peak voxels of abnormal regions as spherical centers, spherical ROIs were constructed around these abnormal regions (with a 6 mm radius). To assess the relationship between the behavioral scores (include MMSE, MoCA and GDS scores) and metrics in these abnormal regions, we used Partial correlation analysis that controlled for age, sex and education by SPSS software (version 20.0; IBM, Chicago, IL, United States). Statistical significance was defined as *p* < 0.05. To control for false positives from multiple comparisons, we used the false discovery rate (FDR) correction in which the *p*-values were multiplied by the number of comparisons.

## Results

### Neuropsychological Results

Demographic and clinical characteristics of 76 patients with MCI, 49 MCIND (19 men; mean age, 65.88 ± 9.762 years) and 27 MCID (11 men; mean age, 63.44 ± 10.58 years) are listed in Table 1. No significant differences were found (*p* > 0.05) in gender, age, education level, and MMSE, MoCA scores between the MCID group and MCIND group. Detailed demographics and the psychological characteristics of the two groups are shown in Table 1.

**TABLE 1:** Demographic and neuropsychological data.

	**MCIND (*n* = 49)**	**MCID (*n* = 27)**	***p***
Age (y, mean ± SD)	65.88 ± 9.762	63.44 ± 10.58	0.2766*^*t*^*
Gender (M/F)	19/30	11/16	0.053*^χ^*
Education (y, mean ± SD)	9.8 ± 3.563	9.444 ± 3.105	0.5412*^*t*^*
MMSE (mean of all points ± SD)	25.43 ± 3.506	25.04 ± 4.052	0.6492*^*t*^*
MoCA (mean of all points ± SD)	19.92 ± 3.416	19.78 ± 4.917	0.8166*^*t*^*
GDS (mean of all points ± SD)	5.66 ± 2.847	15.44 ± 4.635	<0.0001*^*t*^*

*MCID, mild cognitive impairment with the symptom of depression; MCIND, non-depressed mild cognitive impairment; MMSE, Mini Mental State Examination; MoCA, Montreal Cognitive Assessment; GDS, Geriatric Depression Scale; SD, standard deviation. χ, the *p*-value was obtained by the chi-square test; *t*, The *p*-value was obtained by the two-sample *t*-test.*

### Alterations of Region IBA Changes Between MCID and MCIND

#### The Comparison of ALFF

There were no significant differences in ALFF between the MCID group and MCIND group.

#### The Comparison of fALFF

**FIGURE 1:**
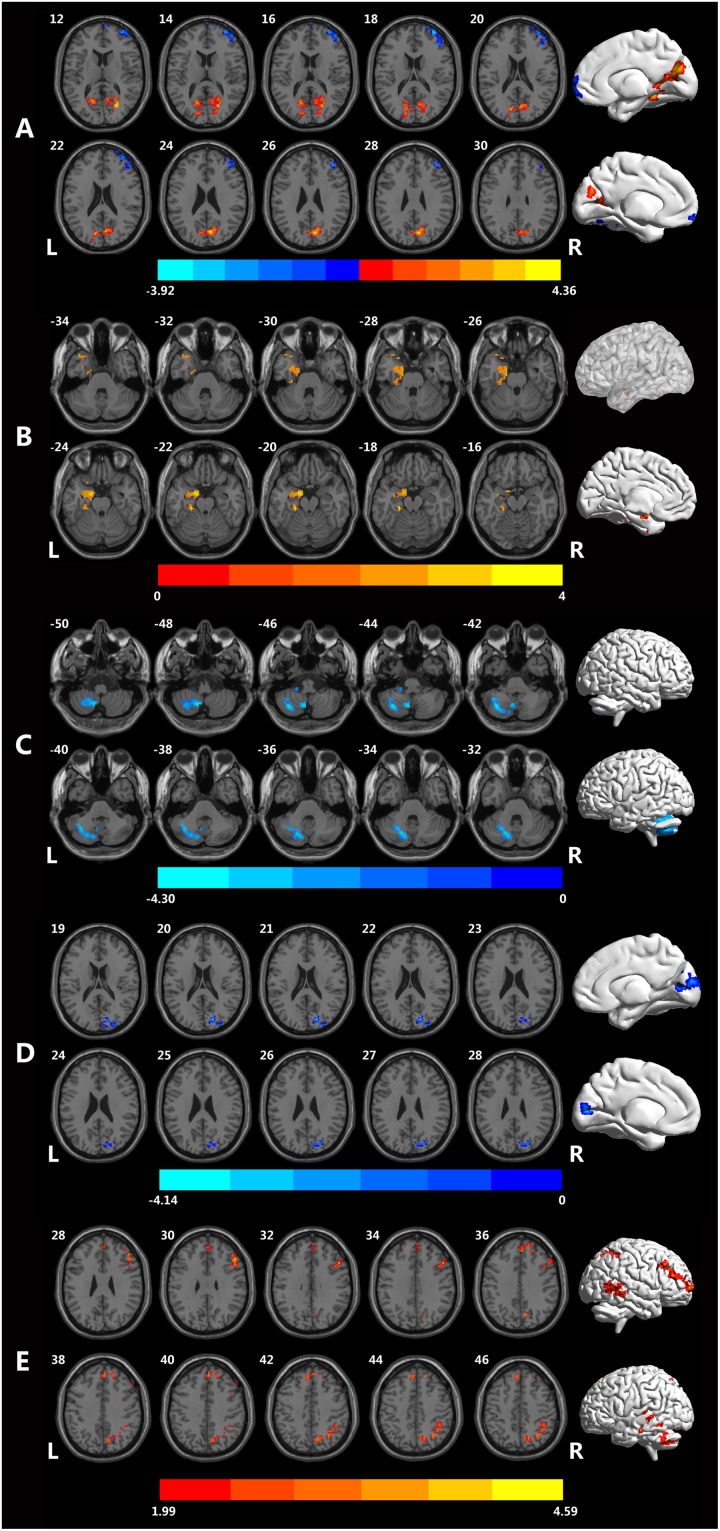
Brain regions showing different rsfMRI values between the MCID and MCIND groups. **(A)** Brain regions with significant differences in fALFF between the MCID group and MCIND group. **(B)** Brain regions with significant differences in PerAF between the MCID group and MCIND group. **(C)** Brain regions with significant differences in ReHo between the MCID group and MCIND. **(D)** Brain regions with significant differences in dfALFF between the MCID group and MCIND group. **(E)** Brain regions with significant differences in dReHo between the MCID group and MCIND group (after GRF correction; voxel-wise *p* < 0.05, cluster-wise *p* < 0.05, two-tailed). The color bar indicates the *T*-value.

As shown in the Figure 1, for fALFF, MCID decreased in inferior temporal gyrus (ITG), middle temporal gyrus (MTG), middle frontal gyrus, inferior occipital gyrus, and cerebellum, but increased in cuneus, calcarine, lingual. The significant differences in fALFF between the two groups are shown in Table 2 and Figure 1A.

**TABLE 2:** Brain regions with significantly differences rsfMRI values in the MCID group compared with the MCIND group.

**Measurements**	**Brain regions**	**MNI coordinates**			**Voxles**	***T*-value**
		**x**	**y**	**z**		
fALFF	Cerebellum Posterior Lobe	−15	−54	−57	339	−3.9226
	Middle Temporal Gyrus_L	−57	−57	0	241	−3.8508
	Precuneus_R	24	−48	3	459	4.3631
	Middle Frontal Gyrus_R	36	57	18	230	−3.6827
PerAF	Parahippocampus_L	−24	−30	−18	287	4.2358
ReHo	Left Cerebellum	−9	−60	−48	571	−4.2962
	Opercular part of inferior frontal gyrus_R	48	21	33	93	4.019
	Superior Frontal Gyrus_R	3	42	36	79	3.3601
	Inferior Parietal Lobule_R	42	−54	48	176	3.7523
dfALFF	Middle Occipital Gyrus, Cuneus, Calcarine, right Superior occipital gyrus	18	−81	21	218	−4.1399
dReHo	Left Cerebellum Posterior Lobe	−36	−63	−39	92	4.4288
	Middle Temporal Gyrus, Inferior Temporal Gyrus	−54	−45	−12	87	4.2681
	Medial Frontal Gyrus, Superior Frontal Gyrus	15	69	3	175	3.501
	Superior Temporal Gyrus, Middle Temporal Gyrus	63	−39	6	84	3.7049
	Middle Frontal Gyrus, opercular part of inferior frontal gyrus, Right Cerebrum	48	21	33	93	4.019
	Medial Frontal Gyrus, Superior Frontal Gyrus	3	42	36	79	3.3601
	Inferior Parietal Lobule, Superior Parietal Lobule, Precuneus	42	−54	48	176	3.7523
	Middle Temporal Gyrus_L	−57	−57	0	241	−3.8508
	Precuneus_R	24	−48	3	459	4.3631
	Middle Frontal Gyrus_R	36	57	18	230	−3.6827

#### The Comparison of PerAF

We found that in MCID group, PerAF increased in left parahippocampus gyrus and temporal gyrus. The significant differences in PerAF between the MCID and MCIND group are shown in Table 2 and Figure 1B.

#### The Comparison of ReHo

Using ReHo, there were only a small amount of group differences between MCID and MCIND group in cerebellum. More details were shown in the Table 2 and Figure 1C.

#### The Comparison of dALFF

There were no significant differences in dALFF between the MCID group and MCIND group.

#### The Comparison of dfALFF

As shown in the Figure 1, for dALFF, compared to MCIND group, MCID group showed decreased dALFF in the bilateral cuneus, middle occipital gyrus, right superior occipital gyrus and calcarine. The significant differences in dfALFF between the MCID group and MCIND group are shown in Table 2 and Figure 1D.

#### The Comparison of dReHo

Using dReHo, compared to MCIND group, MCID group exhibited obvious increase in bilateral MTG, ITG, superior temporal gyrus (STG), superior parietal lobule, inferior parietal gyrus (IPG), precuneus, superior frontal gyrus (SFG), middle frontal gyrus (MFG), opercular part of inferior frontal gyrus (IFG) and right cerebellum. More details were shown in the Table 2 and Figure 1E.

### Correlational Analysis

There was no significant correlation between clinical behavioral scores and any rsfMRI metrics.

## Discussion

Most previous studies have focused on the depression-related or MCI-related brain functional changes. However, the brain function in MCI patients combined with depression is still uncertain. In the current study, we observed alterations in IBA during the resting state in 7 resting state parameters, including four static measurements and three dynamic characteristics in MCID and MCIND patients. We found several brain regions especially in frontal gyrus, temporal gyrus and parahippocampus gyrus, showed significant differences in fALFF, PerAF, ReHo, dfALFF and dReHo between the MCID and MCIND groups. These findings may develop a better understanding of the relationship between depressive symptoms and memory deficits.

As we supposed, the differences of abnormal spontaneous brain activity between MCID and MCIND patients could be distinguished by rsfMRI. We found significant differences between MCID and MCIND group in frontal gyrus [included superior frontal gyrus (SFG), middle frontal gyrus (MFG) and inferior frontal gyrus (IFG)], temporal gyrus (included MTG, ITG and STG), hippocampus, parahippocampus gyrus, IPG and cuneus. The robustness of the results was tested to prove the regions we found was stability and repeatability (Supplementary Figures S2, S3). In the studies pretend to explore the neural mechanism about MCI, AD or depression, these regions also have been mentioned (Langenecker et al., 2007; Alexopoulos et al., 2008; Yao et al., 2009; Lee et al., 2012; Xie et al., 2012; Zhang et al., 2012; Rizio and Dennis, 2014; Guo et al., 2016; Stoyanov et al., 2017; Kandilarova et al., 2018; Yang et al., 2018; Kandilarova et al., 2019). These brain regions may be the proof of a possible shared pathophysiology in both depression and MCI (Yao et al., 2009; Zhang et al., 2012). Other studies found that depressive symptoms in AD had biochemical manifestations similar to depression, suggesting that they might share a common pathway at the biochemical level, this phenomenon may be same in those MCI (Langenecker et al., 2007; Alexopoulos et al., 2008; Lee et al., 2012; Xie et al., 2012; Taylor et al., 2013; Rizio and Dennis, 2014). In studies focused on brain structure found that depression may cause structural changes in frontal and temporal regions (Koolschijn et al., 2009; Kandilarova et al., 2019). These findings suggested that a circuit may be involved in the frontal cortex is associated with the functional neuroanatomy of depression (Langenecker et al., 2007; Yang et al., 2016).

Meanwhile, regions we found the IBA changes mostly related to default mode network (DMN) and executive control network (ECN). Executive functions are control mechanisms that adjust aspects of emotion and cognition, and disruption to these processes is related to worse clinical prognosis of depression (Morimoto et al., 2015). According to previous neuroimaging studies, the DMN s linked to self-referential thought, and the episodic memory retrieval and scene construction (Lei et al., 2013). It is supported by results from previous studies about late-life depression (LLD). LLD usually has a hyperactive DMN (increased rumination, defected cognition) and a hypoactive ECN (low cognitive control associated with emotional response), which may reflect clinical features of depressive symptoms (Aizenstein et al., 2014; Karim et al., 2017).

Above all the parameters we estimated, we didn’t find significant differences in ALFF or dALFF. It has been pointed out that the ALFF could be influenced by the physiological noise irrelevant to brain activity, and dALFF has a temporal variability related to specific topographic (Zou et al., 2008; Liao et al., 2019). As for fALFF, it could effectively suppress the physiological noise, but is not as stable as ALFF in gray matter regions (Zou et al., 2008; Zuo et al., 2010). We firstly used PerAF to estimate IBA of MCID patients, in parahippocampus, MCID had an increased PerAF. Previous study by using both ALFF and PerAF found that the ALFF was similar to PerAF, but PerAF was better than ALFF in inter-scanner reliability (Zhao et al., 2018). Although it is still unclear what causes the difference between those parameters and the potential physiological significance might be, with the limitation of each parameters, it is still necessary to take into consideration all these metrics (Zuo et al., 2010). We found ReHo only decreased in cerebellum, while another study found that MCID patients showed significantly higher coherence ReHo (cReHo) than MCIND patients in the left Heschl’s gyrus and thalamus, lower cReHo in the left postcentral gyrus (Liu et al., 2019). These differences may be influenced by different calculation of ReHo. Also, the recruit criteria of MCID group could be another factor. Depression is a highly heterogeneous disease, and there are no clear gold biomarkers for diagnosis (Ismail et al., 2017). In addition, the diagnose of depression syndrome is based on clinical symptoms and a rating scale, which is inevitably subjective. Further, evidence suggests that evaluating depressive symptoms comprehensively and accurately in those with neurocognitive disorders is difficult because of atypical symptoms in elders, and the interaction between depression and cognitive impairment in older adults makes it further complicated (Ting et al., 2010; Ismail et al., 2017). In our study, we selected the GDS to evaluate depressive symptoms in MCI, while other studies have used different scales, such as HAMD and the NPI (Liu et al., 2019).

Interestingly, we found that the results of dynamic measures differ from the static measures, this had also been found in other studies of diseases (Sourty et al., 2016; Cordova-Palomera et al., 2017). We found that fALFF in MCID decreased in temporal gyrus (ITG, MTG), MFG and inferior occipital gyrus, but increased in cuneus, calcarine, lingual, while in d fALFF, we found an increase in the cuneus, middle occipital gyrus. Also, the ReHo doesn’t change in line with dReHo. As for dReHo, compared to MCIND group, MCID group exhibited an obvious increase in temporal gyrus (MTG, ITG, STG), frontal gyrus (MFG, SFG, IFG) and right cerebellum. However, using ReHo, we only found differences in cerebellum. Sourty et al. (2016) implied that some brain function alterations in Lewy body dementia can be detected utilizing dynamic FC but not static FC by sliding-window analysis (Sourty et al., 2016). The concept of dynamic neuroimage characteristics may provide a proper way to summarize changes in spatial patterns over time and to track differences in disease (Calhoun et al., 2014).

This study had several potential limitations. Firstly, this is a cross-sectional study, we did not investigate the conversion of MCID/MCIND to AD. These two sub-types of MCI patients may have different disease progress into AD. We will focus on not only the different clinical symptoms and their different brain areas, but also the potential different follow up consequences for MCID and MCIND patients. Secondly, it remains unclear that the mechanism of the differences and abnormalities in the patients of MCID and MCIND. In further studies, we would like to combine the rsfMRI, structural MRI and other biophysical data simultaneously with a larger sample and would reveal structural and biological substrates underlying these functional deficits in MCI and MCID patients.

## Conclusion

In summary, we have investigated the IBA of MCID and MCIND using rsfMRI technique. We have found some obvious difference in the IBA between MCID and MCIND in the regions such as frontal gyrus (included SFG, MFG and IFG), temporal gyrus (included MTG, ITG and STG), parietal gyrus (superior parietal gyrus, IPG), occipital gyrus, parahippocampus gyrus and cuneus. The rsfMRI study suggests that the abnormal IBA pattern of the whole-brain functional activity may serve as an early biomarker for the detection of cognitive deficits and emotional problems in MCI patients.

## Author Contributions

YY analyzed the data and wrote the manuscript. ZL analyzed the data. YL, JY, GP, and KZ evaluated the subjects and collected the data. XJ and BL designed the experiment and monitored the quality of the experiment.

## Conflict of Interest

The authors declare that the research was conducted in the absence of any commercial or financial relationships that could be construed as a potential conflict of interest.

## Footnotes

1 http://www.fil.ion.ucl.ac.uk/spm

2 http://www.restfmri.net/forum/
