# Supplementary material for: Depression Affects Intrinsic Brain Activity in Patients With Mild Cognitive Impairment
Source: Front Neurosci. 2019 Dec 17;13:1333. doi: 10.3389/fnins.2019.01333 (PMC6928005; doi:10.3389/fnins.2019.01333)
Supplement: Supplementary file 1 [file Data_Sheet_1.docx]

**Supplementary Methods**

**Validation Analysis**

To validate the variability of our findings, we carried out a leave-one-out validation to test the reproducibility and robustness of the previous findings. For the leave-one-out procedure, each time randomly leaving a different MCID subject out, to compare against the whole MCIND group. This procedure was repeated, and each time leaving a different subject out in MCID group. Then, the overlap map of these group comparison maps was compiled to describe the robustness of the main results (Esterman et al., 2010; Li et al., 2015a; Li et al., 2015b).

Results

Neuropsychological Results

Demographic and clinical characteristics of 27 MCID (11 men; mean age, 63.44 ± 10.58 years) and 50 NC (14 men; mean age, 66.02 ± 9.614 years) are listed in Table 1. No significant differences were found (*p* > 0.05) in gender, age between the MCID and NC group. The education level, and MMSE, MoCA and GDS scores were found different in the two groups. Detailed demographics and psychological characteristics of the MCID patients and NC are shown in Table S1.

Table S1 Demographic and neuropsychological data

|  | MCID (n = 27) | NC (n = 50) | *p* |
| --- | --- | --- | --- |
| Age (y, mean ± SD) | 63.44 ± 10.58 | 66.02 ± 9.614 | 0.2812*^t^* |
| Gender (M/F) | 11/16 | 14/36 | 0.2546*^χ^* |
| Education (y, mean ± SD) | 9.444 ± 3.105 | 11.26 ± 3.773 | 0.0101*^t*^* |
| MMSE (mean of all points ± SD) | 25.04 ± 4.052 | 27.88 ± 1.945 | 0.0002*^t*^* |
| MoCA (mean of all points ± SD) | 19.78 ± 4.917 | 25.3 ± 2.533 | < 0.0001*^t*^* |
| GDS (mean of all points ± SD) | 15.44 ± 4.635 | 10.19 ± 6.357 | < 0.0001*^t*^* |

Table S1. MCID, mild cognitive impairment with the symptom of depression; NC, normal controls; MMSE, Mini Mental State Examination; MoCA, Montreal Cognitive Assessment; GDS, Geriatric Depression Scale; SD, standard deviation. *χ*, The *p* value was obtained by the chi-square test; *t*, The *p* value was obtained by the two-sample *t* test.

**Alterations of region IBA changes** **between MCID and NC**

**The Comparison of ALFF**

As shown in the FigureS1A, ALFF increased in Middle Occipital gyrus (MOG), while decreased in right supramarginal and precentral gyrus compared with NC.

**The Comparison of fALFF**

For fALFF, MCID decreased in left middle temporal gyrus (MTG), right precentral gyrus, but increased in right calcarine. The significant differences in fALFF between the MCID group and NC group are shown in Table S2 and Figure S1B.

**The Comparison of PerAF**

We found that in MCID group, PerAF decreased in inferior frontal gyrus (IFG), precentral gyrus, middle cingulum, precuneus and left angular. The significant differences in PerAF between the MCID group and NC group are shown in Table S2 and Figure S1C.

**The Comparison of ReHo**

Using ReHo, we found MCID had an increased in right. The significant differences in ReHo between the MCID group and NC group are shown in Table S2 and Figure S1D.

**The Comparison of dALFF**

As shown in the FigureS1E, for dALFF, compared to NC group, MCID group showed increased dALFF in the superior frontal gyrus.

**The Comparison of dfALFF**

As shown in the FigureS1F, for dfALFF, compared to NC group, MCID group showed increased dALFF in MTG, right precentral gyrus. The significant differences in dfALFF between the MCID group and MCIND group are shown in Table 2 and Figure 1F.

**The Comparison of dReHo**

Using dReHo, compared to NC group, MCID group exhibited obvious increase in bilateral MTG, MOG, cerebellum, right precentral gyrus and angular gyrus. More details were shown in the Table 2 and Figure 1G.


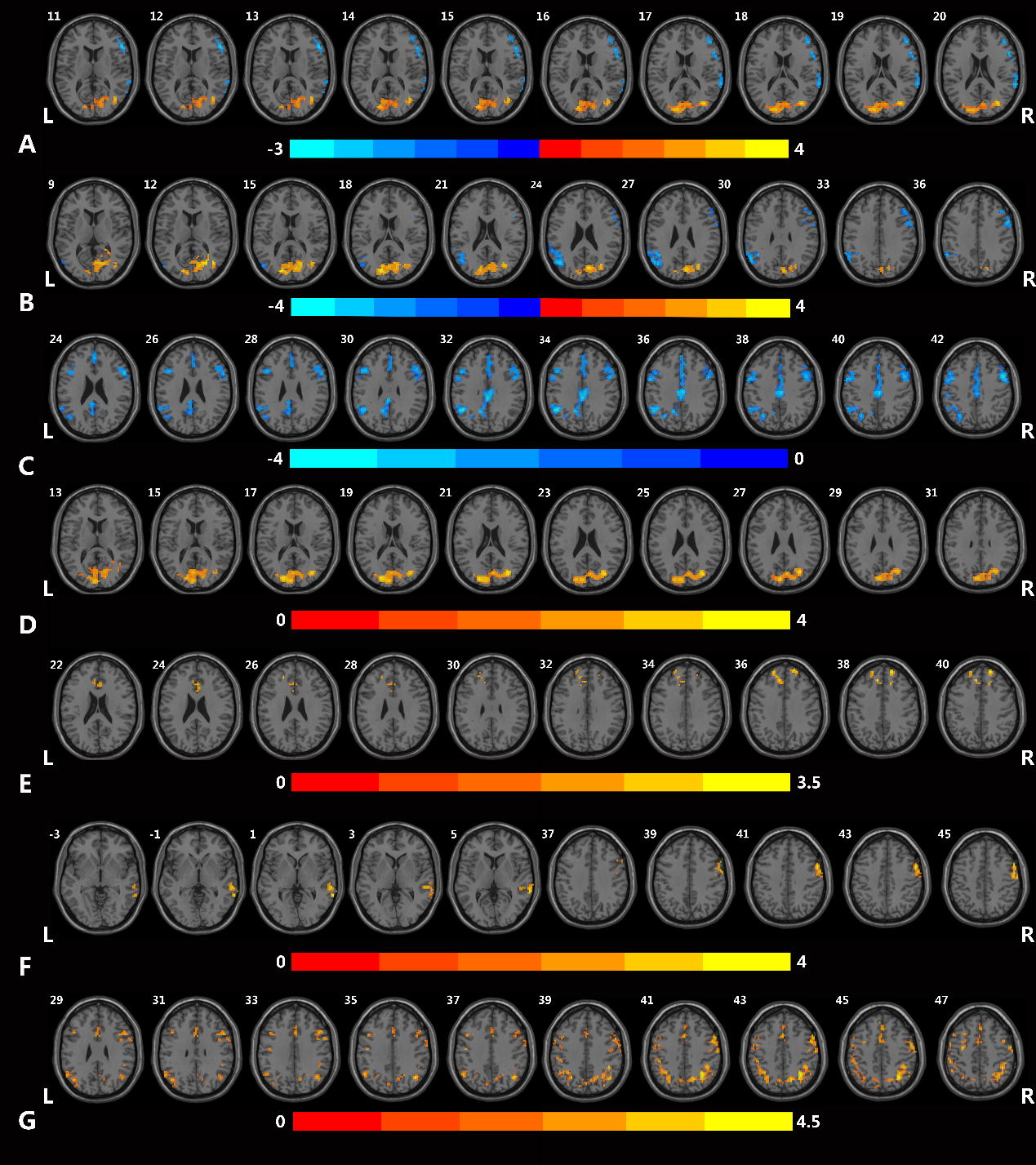


FigureS1 | Brain regions showing different rsfMRI values between the MCID and NC groups. (A) Brain regions with significant differences in ALFF between the MCID and NC. (B) Brain regions with significant differences in fALFF between the MCID and NC. (C) Brain regions with significant differences in PerAF between the MCID and NC. (D) Brain regions with significant differences in ReHo between the MCID and NC. (E) Brain regions with significant differences in dALFF between the MCID and NC. (F) Brain regions with significant differences in dfALFF between the MCID and NC. (G) Brain regions with significant differences in dReHo between the MCID and NC. (after GRF correction; voxel-wise *p* < 0.05, cluster-wise *p* < 0.05, two-tailed). The color bar indicates the *T* value.

Table S2. Brain regions with significantly differences rsfMRI values in the MCID group compared with the NC group

| Measurements | Brain regions | MNI coordinates | | | Voxles | *T* value |
| --- | --- | --- | --- | --- | --- | --- |
|  |  | x | y | z |  |  |
| ALFF | Occipital_Mid_R | 33 | -75 | 21 | 759 | 4.7368 |
|  | SupraMarginal_R | 57 | -48 | 30 | 300 | -3.6856 |
|  | Precentral_R | 48 | 6 | 42 | 277 | -3.9959 |
| fALFF | Calcarine_R | 27 | -69 | 12 | 754 | 4.055 |
|  | Temporal_Mid_L | -45 | -66 | 24 | 256 | -4.1244 |
|  | Precentral_R | 48 | 9 | 42 | 590 | -4.5854 |
| PerAF | Frontal_Inf _L | -48 | 18 | 30 | 587 | -3.543 |
|  | Precentral_R, Frontal_Inf_Tri_R | 48 | 6 | 42 | 588 | -4.4137 |
|  | Cingulum_Mid_R, Precuneus | 3 | -21 | 36 | 788 | -3.8737 |
|  | Angular_L | -45 | -54 | 33 | 300 | -4.0419 |
| ReHo | Occipital_Mid_R | 33 | -75 | 21 | 1299 | 4.4716 |
| dALFF | Frontal_Sup_R | 15 | 51 | 42 | 291 | 3.7344 |
| dfALFF | Temporal_Mid_R | 66 | -45 | 0 | 91 | 4.2152 |
|  | Precentral_R | 54 | 6 | 42 | 105 | 4.0625 |
| dReHo | Cerebelum_Crus1_R | 33 | -78 | -33 | 137 | 3.4313 |
|  | Cerebelum_Crus1_L | -30 | -78 | -33 | 110 | 4.2075 |
|  | Temporal_Mid_L | -65 | -53 | -9 | 80 | 3.4166 |
|  | Temporal_Mid_R | 69 | -39 | 9 | 198 | 4.0516 |
|  | Occipital_Mid_L | -33 | -87 | 21 | 145 | 4.4828 |
|  | Occipital_Mid_L | -27 | -63 | 39 | 230 | 4.0199 |
|  | Angular_R | 42 | -63 | 36 | 419 | 4.7496 |
|  | Precentral_R | 57 | 12 | 42 | 159 | 3.7198 |
|  | Cingulum_Mid_L | 0 | 12 | 42 | 124 | 3.8806 |
|  | Frontal_Sup_L | -27 | -3 | 63 | 169 | 3.9613 |

**Results of Validation Analysis**

**Validation Analysis**

We validated our main findings by leave-one-out validation. The leave-one-out results showed that our main findings were highly reproducible, the spatial patterns of all the metrics are validity and reliability. Figure S2 shows the overlap of MCID and MCIND. Figure S3 shows the overlap of MCID and NC.


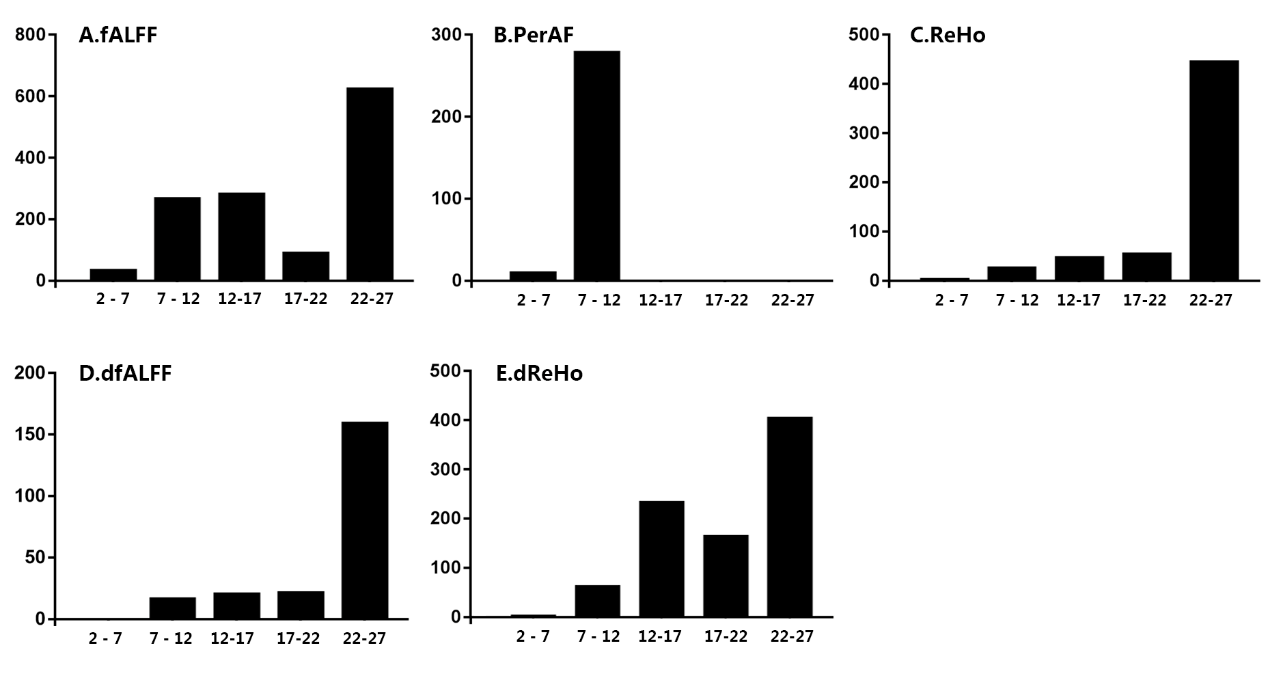


Figure S2 |The leave one out results showed the representative cross-sections of the validation of the regional IBA differences between MCID and MCIND detected by different measurements.

Analyses procedure: github: https://github.com/Liziqi-code/Liziqi


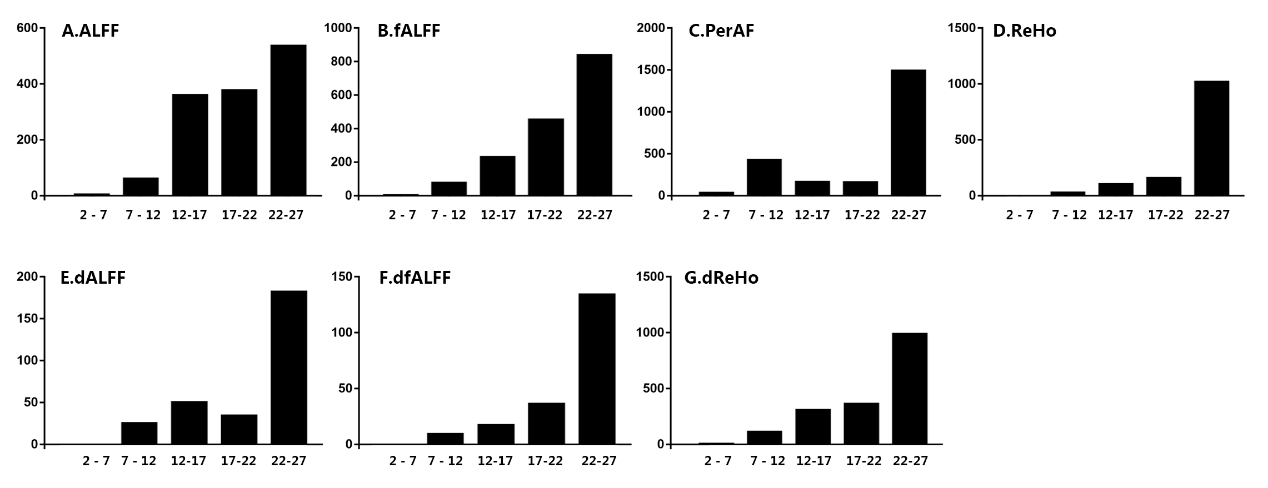


Figure S3 |The leave one out results showed the representative cross-sections of the validation of the regional IBA differences between MCID and NC detected by different measurements
